# Patient and family co-developed participant information to improve recruitment rates, retention, and patient understanding in the Rehabilitation Strategies Following Oesophago-gastric and Hepatopancreaticobiliary Cancer (ReStOre II) trial: Protocol for a study within a trial (SWAT)

**DOI:** 10.12688/hrbopenres.12950.2

**Published:** 2020-11-10

**Authors:** Linda O'Neill, Peter Knapp, Suzanne L. Doyle, Emer Guinan, Adwoa Parker, Ricardo Segurado, Deirdre Connolly, Jacintha O'Sullivan, John V. Reynolds, Juliette Hussey

**Affiliations:** 1 Discipline of Physiotherapy, School of Medicine, Trinity College Dublin, University of Dublin, Dublin, Ireland; 2 Department of Health Sciences and the Hull York Medical School, University of York, York, UK; 3 School of Biological and Health Sciences, Technological University Dublin, Dublin, Ireland; 4 School of Medicine, Trinity College Dublin, University of Dublin, Dublin, Ireland; 5 School of Public Health, Physiotherapy and Sport Sciences, University College Dublin, Dublin, Ireland; 6 Discipline of Occupational Therapy, School Medicine, Trinity College Dublin, University of Dublin, Dublin, Ireland; 7 Department of Surgery, Trinity Translational Medicine Institute, Trinity College Dublin, University of Dublin, and St James's Hospital, Dublin, Ireland

**Keywords:** SWAT, participant information, recruitment, retention, trial understanding, public and patient involvement

## Abstract

**Background:** Whilst the potential benefits of exercise rehabilitation in cancer survivorship are plentiful, recruitment to survivorship rehabilitation trials remains suboptimal. There is growing evidence that Public and Patient Involvement (PPI) initiatives can increase the rate of recruitment to research. This study within a trial (SWAT) will examine if participant information co-developed by patients and their families can lead to greater recruitment rates, retention and understanding of the Rehabilitation Strategies in Oesophago-gastric and Hepatopancreaticobiliary Cancer (ReStOre II) trial when compared to standard participant information.

**Methods:** This SWAT will be carried out over two phases. Phase I will utilise qualitative methods to develop (Phase Ia) and refine (Phase Ib) the new participant information. Phase Ia will recruit up to 20 survivors of upper gastrointestinal or hepatopancreaticobiliary cancer, or their family members, to take part in a focus group or interview to develop the new participant information. Focus groups/interviews will be recorded, transcribed verbatim and analysed thematically. In Phase Ib, participants will return for a second focus group/interview to refine the participant information. Once finalised, the participant information will be submitted to ethics for approval. In Phase II, potential participants for the ReStOre II trial will be randomly assigned to receive either the standard or patient and family co-developed participant information. The two forms of participant information will be compared by recruitment and retention rates, and participant understanding of the trial (Decision-Making Questionnaire).

**Discussion:** We anticipate that engaging with patients and their families to develop participant information will help to increase patient understanding of the ReStOre II trial and therefore recruitment and retention rates. The results of this SWAT will indicate the usefulness of this strategy for optimising recruitment to exercise rehabilitation trials in cancer survivorship.

**Registration: **SWAT: Northern Ireland Hub for Trials Methodology Research SWAT Repository Store ( SWAT-100). ReStOre II: ClinicalTrials.gov ( NCT03958019).

## Introduction

As cancer survival rates continue to improve, optimising survivorship care has become a national priority in the Republic of Ireland ^1– 3^. Exercise rehabilitation is a care strategy with considerable potential to optimise physical function and quality of life in cancer survivorship ^4^. However, recruitment and retention in cancer exercise trials remains a challenge ^5^, which may be detrimental to the validity of trial results. Accordingly, there is strong rationale to investigate strategies which may aid recruitment and retention to cancer exercise trials.

Public and Patient Involvement (PPI) has been described as research being carried out with or by members of the public rather than to, about, or for them ^6, 7^. This approach to research is encouraged as it is felt that those affected by research should have a say in how it is carried out ^8^. There is also evolving evidence that PPI can increase the rate of recruitment to research and improve its quality and impact ^9^. A recent systematic review and meta-analysis by Crocker *et al.* ^6^ investigating the impact of PPI on patient enrolment and retention in clinical trials demonstrated that PPI significantly increased the odds of participant recruitment (odds ratio 1.16, 95% confidence interval and prediction interval 1.01 to 1.34). An example of a PPI strategy to enhance trial enrolment is the inclusion of patients and the public in the design of participant information. Traditional participant information has consistently been criticized for being too lengthy, using technical or difficult language, and for lacking navigability and visual appeal ^10^. Furthermore, it is reported that patients with cancer may gain little understanding of the risks and benefits of research from provided participant information ^11^. Therefore, participant information may in fact become a barrier to trial understanding and enrolment, and there is therefore considerable rationale to optimise trial participant information. 

The Rehabilitation Strategies following Oesophago-gastric and Hepatopancreaticobiliary cancer (ReStOre II) trial (ClinicalTrials.gov Identifier: NCT03958019) will examine by randomised controlled trial (RCT) a multidisciplinary rehabilitation programme (ReStOre II) for survivors of cancer of the oesophagus, stomach, pancreas, and liver. The ReStOre II programme will consist of supervised and self-managed exercise, 1:1 dietary counselling, and education sessions. In a previous pilot RCT, this programme was found to lead to significant improvements in cardiorespiratory fitness ^12^, and positively impact on physical, mental and social wellbeing ^13^. Furthermore, a patient recruitment rate of 40% was achieved ^12^. Whilst this rate is higher than rates cited by other cancer rehabilitation programmes (11.1%) ^14^, given the potential benefits of participation there is just cause to attempt to achieve greater rates of enrolment for ReStOre II. Importantly, an increased recruitment rate would accelerate the progress, completion and dissemination of the ReStOre II trial. To this end, this study within a trial (SWAT) will engage with patients and their families and ask them to contribute to the development of participant information and examine its impact by an embedded randomised controlled trial.

### Study aims

This SWAT aims to examine within the ReStOre II RCT if participant information co-developed by patients and their families can lead to improved recruitment rates, retention, and participant understanding of the study in comparison to standard participant information leaflets.

Specific objectives are:

- To engage with patients with upper gastrointestinal (UGI) or hepatopancreaticobiliary (HPB) cancer, namely oesophageal/gastric/pancreatic/liver cancer and their family members to develop participant information for the ReStOre II RCT.
- To examine the impact of the patient and family co-developed participant information on ReStOre II recruitment rates.
- To determine the impact of the patient and family co-developed participant information on ReStOre II retention rates.
- To explore the impact of the patient and family co-developed participant information on patients’ understanding of the ReStOre II trial.

## Methods

### Study design

**Figure 1.**
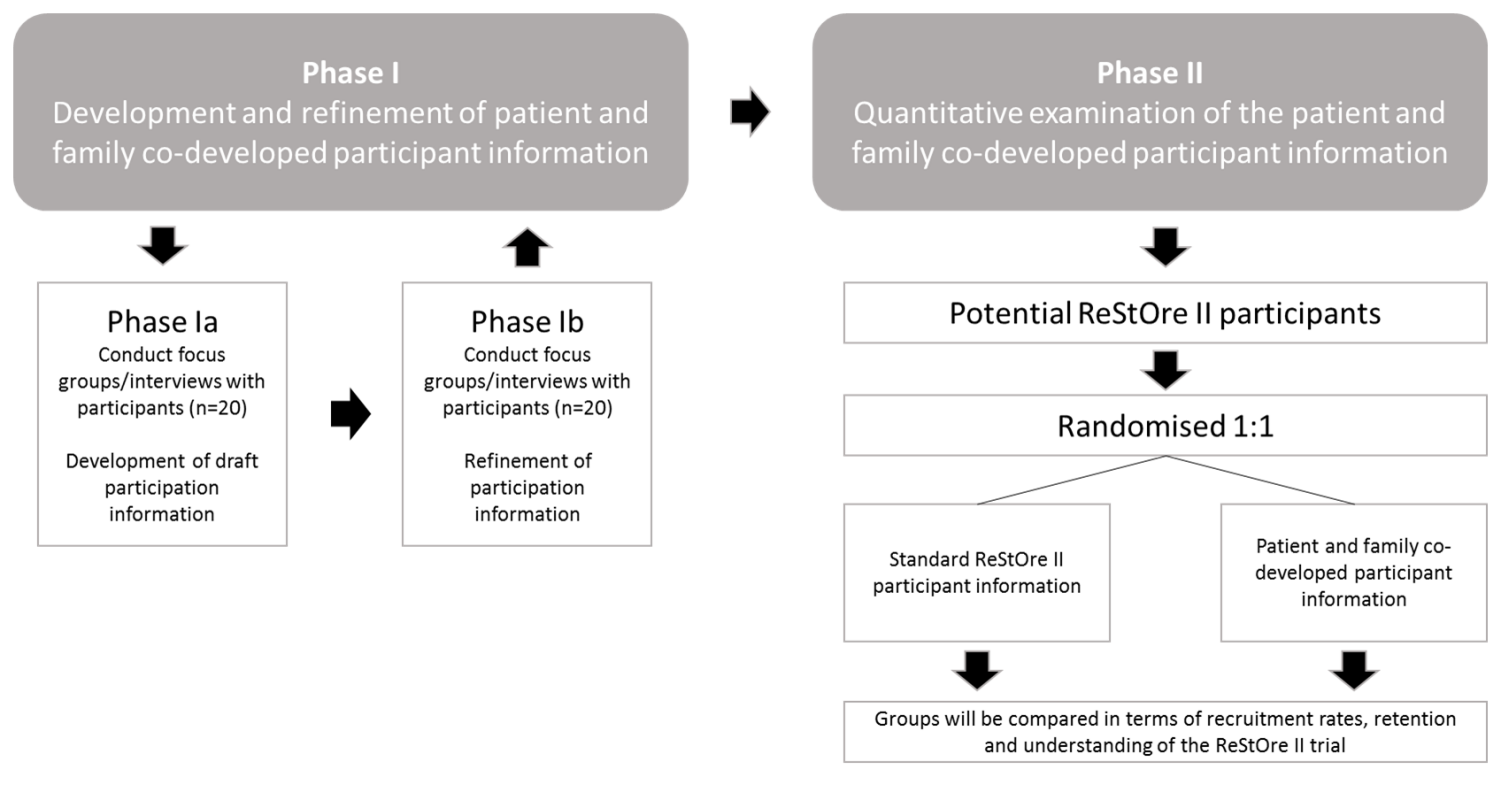
Study design. ReStOre, Rehabilitation Strategies Following Oesophago-gastric and Hepatopancreaticobiliary Cancer.

The study is divided into two phases; Phase I (development) and Phase II (evaluation). The study design is presented in Figure 1. Phase I will utilise qualitative methods to develop and refine the patient and family co-developed participant information. In Phase II, the patient and family co-developed participant information will be compared to standard participation information by a randomised controlled trial embedded within the ReStOre II randomised controlled trial. 

### Study participants

Phase I of the SWAT will recruit patients who have previously undergone surgery for cancer of the oesophagus, stomach, liver, or pancreas, and/or the spouses, partners or close relatives of these patients. Individuals with communication or cognitive difficulty that would impair their ability to take part in a semi-structured focus group/interview will be excluded. Twenty participants will be recruited to ensure a diverse range of views are obtained. Multiple focus groups/ interviews will be held ensuring there are no more than 12 participants per focus group to facilitate in depth discussion. The sample of patients will include men and women, of a range of ages, cancer types and educational backgrounds. Three strategies will be utilised for recruitment: i) previous participants of the ReStOre I trial will be sent a letter and participation leaflet inviting them and their partner/spouse/close relative to participate; ii) participant information leaflets will be supplied to patients attending the UGI cancer clinic at St James’s Hospital (SJH); and iii) potential participants may make themselves known to the research team by replying to adverts disseminated through our charity partners, the Oesophageal Cancer Fund and the Irish Cancer Society. Individuals who are willing to participate in Phase I will be required to give written informed consent for participation and data processing.

Phase II of the SWAT will involve potential ReStOre II RCT participants. The ReStOre II RCT will recruit patients with cancer of the oesophagus/stomach/liver or pancreas, who have completed curative treatment, from three hospitals in Dublin, Ireland; SJH, St Vincent’s University Hospital, and Tallaght University Hospital (TUH). Participants must be ≥ three months post oesophagectomy, total gastrectomy, pancreaticoduodenectomy, or major liver resection (+/- neoadjuvant/adjuvant chemotherapy/chemoradiotherapy) and any adjuvant treatment must be complete. Exclusion criteria includes evidence of ongoing serious post-operative morbidity, evidence of active or recurrent disease or any contraindication to maximal exercise testing.

### The SWAT

***Phase I.*** Phase I of the SWAT will be carried out over two sub-phases: Phase Ia (development of the patient and family co-developed participant information) and Phase Ib (refinement of the patient and family co-developed participant information).

***Phase Ia - Development of patient and family co-developed participant information.*** In Phase Ia, survivors of UGI and HPB cancer and spouses, partners, and close family members of these patients will be invited to participate in a focus discussion group or individual interview study, as preferred. The focus groups and interviews will take place in the Clinical Research Facility at SJH. Individuals who consent to participation and are unable to attend SJH at the time of the focus group will be offered the opportunity to take part in a 1:1 interview at SJH or via phone or Skype. The focus group or interview will be led by a qualitative researcher experienced in PPI initiatives. The focus group discussion and interviews will be audio recorded. Using an agreed topic guide ^15^, participants will be invited to discuss participant information development, by asking them to comment on the participant information leaflet developed for the ReStOre II trial and make suggestions for amending and enhancing it. The Consensus-Orientated-Decision-Making (CODM) model ^16^ will be used to guide the group to reach a consensus.

The CODM model steps include:

1. Framing the topic
2. Open discussion
3. Identifying underlying concerns
4. Collaborative proposal building
5. Choosing a direction
6. Synthesizing a final proposal
7. Closure

The discussion will also focus on core aspects of information utility and quality: content, language, structure, navigation, and visual impact.

Focus group/interview recordings will be transcribed verbatim. A basic thematic analysis will be undertaken to inform the revision of participant recruitment materials. This will be guided by the Braun and Clarke ^17^ model and using an information quality framework of: content, language, structure, navigation, and visual impact. Researchers will then use the focus group/interview findings to develop an initial draft of the revised patient and family co-developed participant information, a form of ‘participatory design’ ^18, 19^.

***Phase Ib – Refinement of the patient and family co-developed participant information.*** In Phase Ib, participants from Phase Ia will be invited to return to take part in a second focus group/interview. If insufficient participants from the Phase Ia data collection are available for Phase 1b, we will recruit new participants for Phase Ib by applying the same entry criteria. At this session, participants will comment on the participant information that has been developed following the feedback received in Phase Ia, focusing on its structure, content, language, visual impact and navigation. Similar to Phase Ia, the Phase Ib focus groups and interviews will be audio recorded and then transcribed verbatim and reported using a basic thematic analysis. Researchers will then act on patient and relative feedback to edit different sections of the participant information. At the end of this phase of the study, a final draft of the patient and family co-developed participant information will be approved by the research team and patient group. The resultant final participant information will then be submitted as an amendment to the research ethics application for the ReStOre II trial (TUH/SJH and St Vincent’s Hospital Research Ethics Committees).

**Phase I Data management**

The Data Management Plan ^15^ will outline how research data will be handled during and after the project. All participants will be allocated a unique study code. The key to the study code will be stored securely and separately. All transcripts will be stored in locked filing cabinets, in a locked office in a restricted access building with swipe access. Electronic records will be stored on password protected encrypted devices. 

***Phase II.*** Following ethical approval, the new patient and family co-developed participant information will be tested in a prospective, randomised, single blind, parallel trial design. Potential ReStOre II trial participants will be randomised to receive either the standard participant information (control group) or the patient and family co-developed participant information (intervention group) when initially approached for recruitment. Patients will be approached for recruitment at the upper gastrointestinal cancer clinics at SJH, SVUH, and TUH. If recruitment rates are suboptimal, the study will also be advertised through the social media platforms of our charity partners, the Irish Cancer Society and the Oesophageal Cancer Fund.

**Randomisation**

The type of information leaflet each potential participant will be sent will be determined by random allocation. Randomisation will be overseen by the Clinical Research Facility at SJH. Potential participants will be randomised in a 1:1 ratio. Block randomisation with random varying block sizes will be used, with the block sizes specified by the Clinical Research Facility and not shared with other researchers. The allocation lists will be generated by a randomisation system and shared and accessed only by an independent member of the research team not involved in recruitment, to achieve concealment of allocation.

**Blinding**

Potential participants invited to participate in ReStOre II will be blinded to the nature and objectives of the SWAT. The central SWAT team will not be blinded to the allocation of groups but will have no contact with potential patient recruits. Patient recruitment will take place by post or face-to-face. When recruitment happens in-person the recruiting researcher will not be blinded to SWAT allocation if the patient brings the information leaflet that has been given to them; this is unavoidable and we will report the details of the recruitment process so that any potential effect of unblinding can be seen. When recruitment happens in-person we will also ask the recruiting researcher to record which of the two PIS groups they think the patient has been assigned to, as a means of estimating the extent of any unblinding.

**Outcome measures**

The two forms of participant information will be compared in terms of participant recruitment rates (primary outcome), defined as the proportion of participants in each intervention group that are randomised into ReStOre II.

The secondary outcome will be understanding of the trial, which will be assessed using the TRECA Decision-Making Questionnaire (DMQ) ^15^. The DMQ was developed within the TRECA study, which is evaluating digital information about trials for children and adolescents. The questionnaire asks participants to evaluate various aspects of the information and its utility to inform decisions about trial participation. It includes nine Likert items and three open response items ^20^. One week after receiving the participant information participants will receive a follow-up phone call from a member of the research team to confirm if they are interested/ not interested in participation. Following their decision to decline or accept participation individuals will be asked to complete the DMQ. The DMQ will be posted to individuals and they will be given a stamped addressed envelope to return it to the Research team. 

A further secondary outcome will be retention to the trial, defined as the proportion of randomised participants who participated in the ReStOre II main trial up to and including the first follow-up data collection time point.

**Phase II-Data Management**

Electronic and paper data will be stored securely and safely as outlined above in Phase I. 

**Sample size calculations**

The sample size calculations for the ReStOre II trial have been outlined in the main trial protocol. As is usual with a SWAT, we did not undertake a formal power calculation to determine the sample size ^21^, since the sample size is constrained by the number of patients being approached in the ReStOre II host trial. We anticipate a sample size of approximately 300 patients for the embedded SWAT, which is the number of people who will be approached to participate in the ReStOre II host trial (150 per group). Analysed independently, this sample would give 80% power to detect an improvement in recruitment rates from 40 to 56%. This anticipated recruitment rate was determined from our feasibility work in this area ^12^.

**Analysis plan**

Statistical analysis will comprise evaluation of the impact of the revised information on: i) rates of recruitment to the trial (assessed by odds ratios); ii) questionnaire scores, analysed separately for recruited participants and those who refused ReStOre II participation; and iii) rates of retention in the ReStOre II trial (to the first follow-up data collection time point, assessed by odds ratios). Analyses will be conducted on an intention to treat basis, including all randomised participants on the basis of the groups to which they were randomised. Analysis will be conducted using two-sided significance tests at the 5% significance level. For analysis of the primary outcome, logistic regression will be used to produce odds ratios and their associated 95% confidence intervals and p-values having adjusted for the effect of potential confounding variables (e.g. age, sex, cancer type).

### Safety

There are no anticipated harms from taking part in this SWAT. Any incidents/serious incidents that occur will be recorded. Any serious incidents will be reported to the PI and the ethics committee (within 24 hours).

### Trial management and governance

The management of this SWAT will be overseen by the ReStOre II trial management groups; a Trial management Group (TMG), Trial Steering Committee (TSC) and an Independent Data Monitoring Committee (IDMC). The TMG will oversee the daily management of the SWAT. The TSC will meet biannually and provide oversight of the SWAT. The IDMC will also meet biannually and will monitor SWAT data to ensure the safety of the participants. The CRF at SJH will provide independent monitoring and will make reports to the IDMC.

### Dissemination

The results of this SWAT will be disseminated via peer-reviewed publications and conference presentations. Results will also be shared with participants and their families at an education symposium when the study is complete. Upon completion of the trial an anonymised data set will be deposited on a secure online repository in line with open access publication requirements.

### Study status

Recruitment for Phase I began in October 2019.

### Ethical statement

Ethical approval for Phase I has been obtained from TUH/SJH Research Ethics Committee (REC: 2019-09 List 35 (08)). All Phase I participants will be required to give written informed consent. As Phase II is embedded in the multicentre ReStOre II RCT, ethical approval has been sought for Phase II in conjunction with the ReStOre II RCT ethics application from both TUH/SJH REC and St Vincent’s University Hospital REC. Any amendments to the planned protocol will be reported to the ethics committees.

## Discussion

Optimising cancer survivorship care is a health service priority both nationally and internationally ^1, 22^. Given the hypothesised benefits of exercise in cancer survivorship ^4^, and the typical poor accrual rates to cancer exercise trials ^5^, it is imperative to explore strategies to optimise recruitment to such trials. PPI has been employed successfully to optimise recruitment in other clinical populations ^6^; this protocol sets out to examine by a SWAT if patient and family co-developed participant information will have a beneficial impact on recruitment to the ReStOre II trial. If successful, this SWAT will provide a useful template for maximising enrolment to exercise rehabilitation trials and other trials in cancer survivorship.

## Data availability

### Underlying data

All data underlying the results are available as part of the article and no additional source data are required. ****

### Extended data

Open Science Framework: Patient and family co-developed participant information to improve recruitment rates, retention, and patient understanding in the Rehabilitation Strategies Following Oesophago-gastric and Hepatopancreaticobiliary Cancer (ReStOre II) trial: Protocol for a study within a trial (SWAT). https://doi.org/10.17605/OSF.IO/KQXGH ^15^.

This project contains the following extended data:

- 190401 ReStOre II SWAT Focus Group Interview Guide.pdf
- 190401 ReStOre II SWAT Phase I Consent Form.pdf
- 190530 RESTORE II SWAT Decision Making Questionnaire Version 1.pdf
- 190719 Data Management Plan (DMP) Version 1 RESTORE SWAT.pdf

### Reporting guidelines

Open Science Framework: SPIRIT checklist for “Patient and family co-developed participant information to improve recruitment rates, retention, and patient understanding in the Rehabilitation Strategies Following Oesophago-gastric and Hepatopancreaticobiliary Cancer (ReStOre II) trial: Protocol for a study within a trial (SWAT)”. https://doi.org/10.17605/OSF.IO/WH3YM.

Data are available under the terms of the Creative Commons Zero “No rights reserved” data waiver (CC0 1.0 Public domain dedication).
