# Supplementary material for: Patient and family co-developed participant information to improve recruitment rates, retention, and patient understanding in the Rehabilitation Strategies Following Oesophago-gastric and Hepatopancreaticobiliary Cancer (ReStOre II) trial: Protocol for a study within a trial (SWAT)
Source: HRB Open Res. 2020 Nov 10;2:27. Originally published 2019 Oct 28. [Version 2] doi: 10.12688/hrbopenres.12950.2 (PMC7745189; doi:10.12688/hrbopenres.12950.2)
Supplement: Peer review history

# Peer review: Reviewer response for version 2

**DOI:** 10.21956/hrbopenres.14307.r28313

**Published:** 2020-12-08

**Authors:** Shiow-Ching Shun

**Affiliations:** 1 School of Nursing, National Taiwan University, Taiwan, Taiwan

There is no further suggestions for the version 2.

Is the study design appropriate for the research question?

Yes

Is the rationale for, and objectives of, the study clearly described?

Yes

Are sufficient details of the methods provided to allow replication by others?

Yes

Are the datasets clearly presented in a useable and accessible format?

Yes

Reviewer Expertise:

Symptom management in patients with cancer.

I confirm that I have read this submission and believe that I have an appropriate level of expertise to confirm that it is of an acceptable scientific standard.

# Peer review: Reviewer response for version 2

**DOI:** 10.21956/hrbopenres.14307.r28312

**Published:** 2020-11-16

**Authors:** David J. Bowrey

**Affiliations:** 1 Department of Surgery, Leicester Royal Infirmary, University Hospitals of Leicester NHS Trust, Leicester, UK

I can confirm my approval of the reviewers' comments and satisfactory amendments.

Is the study design appropriate for the research question?

Partly

Is the rationale for, and objectives of, the study clearly described?

Yes

Are sufficient details of the methods provided to allow replication by others?

Yes

Are the datasets clearly presented in a useable and accessible format?

Yes

Reviewer Expertise:

Clinical oesophago-gastric surgery, nutrition.

I confirm that I have read this submission and believe that I have an appropriate level of expertise to confirm that it is of an acceptable scientific standard.

# Peer review: Reviewer response for version 1

**DOI:** 10.21956/hrbopenres.14032.r28015

**Published:** 2020-10-15

**Authors:** Shiow-Ching Shun

**Affiliations:** 1 School of Nursing, National Taiwan University, Taiwan, Taiwan

**Is the study design appropriate for the research question?**

 The study design is appropriate for the research question. However, some suggestions are intended to improve the study outcomes. First, the focus groups with 20 participants involve too many persons and it might not have in-depth discussion from the 20 participants. A group with 10-12 participants is suggested. Second, although the authors mentioned that “Twenty participants will be recruited to ensure a diverse range of views are obtained”, the strategy to ensure a diverse range of views has not been addressed. (3) Why the recruitment rates are set from 40 to 56%? References should be cited. (4) The details for analysis plan for logistic regression should be added more details, such as any differences between two groups or confounding factors will be considered.

Is the study design appropriate for the research question?

Yes

Is the rationale for, and objectives of, the study clearly described?

Yes

Are sufficient details of the methods provided to allow replication by others?

Yes

Are the datasets clearly presented in a useable and accessible format?

Yes

Reviewer Expertise:

Symptom management in patients with cancer.

I confirm that I have read this submission and believe that I have an appropriate level of expertise to confirm that it is of an acceptable scientific standard, however I have significant reservations, as outlined above.

# Response

**Published:** 2020-10-29

**Authors:** Linda O'Neill

**Affiliations:** Trinity University, Ireland

Dear Professor Shiow-Ching Shun,

 Many thanks for taking the time to review our protocol paper.

 Please find our response to your comments below.

**The study design is appropriate for the research question. However, some suggestions are intended to improve the study outcomes. First, the focus groups with 20 participants involve too many persons and it might not have in-depth discussion from the 20 participants. A group with 10-12 participants is suggested.** 

*It is now clarified under the Study Participant paragraph that multiple focus groups/ individual interviews will be held. No more than 12 participants will be included per focus group discussions to facilitate in-depth discussion.*

**Second, although the authors mentioned that “Twenty participants will be recruited to ensure a diverse range of views are obtained”, the strategy to ensure a diverse range of views has not been addressed.**  

*An additional sentence has been added to under the Study Participant paragraph to describe the sampling for the qualitative work.*

**Why the recruitment rates are set from 40 to 56%? References should be cited.**

*The anticipated recruitment rate was determined by our feasibility work in this area. This is now cited (reference 12) under the Sample Size Calculation plan.*

**The details for analysis plan for logistic regression should be added more details, such as any differences between two groups or confounding factors will be considered.**

*We have added a sentence to the Analysis Plan section on page 8 to indicate the intended adjustment for possible confounding variables.*

 We hope that you find our responses to your comments satisfactory.

 Kind regards,

 Dr Linda O'Neill

 Project Manager

# Peer review: Reviewer response for version 1

**DOI:** 10.21956/hrbopenres.14032.r27306

**Published:** 2020-04-21

**Authors:** David J. Bowrey

**Affiliations:** 1 Department of Surgery, Leicester Royal Infirmary, University Hospitals of Leicester NHS Trust, Leicester, UK

The trial protocol is very well written and the findings will add to the existing literature.

 I have two points for clarification:

1. In the sample size calculation, a sample of 300 participants is advocated. 
  The RESTORE II trial protocol on the Clinical Trials website lists a sample size of 120 participants. Is the 300 participants correct in the current protocol? Or should it read that 300 eligible patients will be approached in the expectation that 120 will participate in the SWAT (and thus RESTORE II)?
2. The authors indicate that the participants will be blinded to the purpose of the SWAT but that the managing team will not be blinded. This introduces a potentially significant bias, which could undermine the whole methodology of the study, even more so when the primary outcome measure is recruitment rate. It will obviously depend upon who does the recruitment. For this to be scientifically robust, the acceptable approaches could include recruitment performed by a researcher completely separate from the managing team, or blinding the managing team from the arm of trial allocation. Intuitively, the latter seems the better of the two options.

Is the study design appropriate for the research question?

Partly

Is the rationale for, and objectives of, the study clearly described?

Yes

Are sufficient details of the methods provided to allow replication by others?

Yes

Are the datasets clearly presented in a useable and accessible format?

Yes

Reviewer Expertise:

Clinical oesophago-gastric surgery, nutrition.

I confirm that I have read this submission and believe that I have an appropriate level of expertise to confirm that it is of an acceptable scientific standard, however I have significant reservations, as outlined above.

# Response

**Published:** 2020-10-29

**Authors:** Linda O'Neill

**Affiliations:** Trinity University, Ireland

Dear Professor Bowrey, 

 Many thanks for taking the time to review our article. 

 Please find our response to your points below:

1. **The RESTORE II trial protocol on the Clinical Trials website lists a sample size of 120 participants. Is the 300 participants correct in the current protocol? Or should it read that 300 eligible patients will be approached in the expectation that 120 will participate in the SWAT (and thus RESTORE II)?** *To clarify the RESTORE II trial in which this SWAT is embedded will aim to recruit 120 participants. For this SWAT assuming a recruitment rate of 40-56% as per the protocol we anticipate a sample size of approximately 300 patients for the embedded SWAT, which is the total number of people who will be approached to participate in the ReStOre II host trial (150 per SWAT group).*
2. ** The authors indicate that the participants will be blinded to the purpose of the SWAT but that the managing team will not be blinded. This introduces a potentially significant bias, which could undermine the whole methodology of the study, even more so when the primary outcome measure is recruitment rate. It will obviously depend upon who does the recruitment. For this to be scientifically robust, the acceptable approaches could include recruitment performed by a researcher completely separate from the managing team, or blinding the managing team from the arm of trial allocation. Intuitively, the latter seems the better of the two options.•** *We have added two sentences to address this matter to the section on ‘blinding’.*

We hope that you find our responses satisfactory.

 Kind regards,

 Dr Linda O'Neill

 Project Manager
